# Income related inequalities in New Cooperative Medical Scheme: a five-year empirical study of Junan County in China

**DOI:** 10.1186/1475-9276-13-38

**Published:** 2014-05-16

**Authors:** Shasha Yuan, Clas Rehnberg, Xiaojie Sun, Xiaoyun Liu, Qingyue Meng

**Affiliations:** 1 Center for Health Management and Policy, Shandong University, Jinan 250012, China; 2 Center for Health Policy and Management, Institute of Medical Information & Library, Chinese Academy of Medical Sciences & Peking Union Medical College, Beijing 100020, China; 3 Medical Management Center, Karolinska Institutet, SE-17177 Stockholm, Sweden; 4 Peking University China Center for Health Development Studies, Beijing 100191, China

**Keywords:** New Cooperative Medical Scheme, Income related inequality, Concentration index, Inpatient, Outpatient, Medical service utilization, Reimbursement, Out of pocket expenditures, China

## Abstract

**Introduction:**

The Chinese New Cooperative Medical Scheme (NCMS) was launched in 2003 aiming at protecting the poor in rural areas from high health expenditures and improving access to health services. The income related inequality of the reform is a debating and concerning policy issue in China. The purpose of this study is to analyze the degree and changes of income related inequalities in both inpatient and outpatient services among NCMS enrollees from 2007 to 2011.

**Data and methods:**

Data was extracted from the NCMS information system of Junan County in Shandong province from 2007 to 2011. The study targeted all NCMS enrollees in the county, 726850 registered in 2011. Detailed information included demographic data, inpatient and outpatient data in each year. Descriptive analysis of quintiles and standardized concentration index (CI*) were employed to examine the income related inequalities in both inpatient and outpatient care.

**Results:**

For inpatient care, the benefit rate CI* was positive (pro-rich) and increased from 2007 to 2011 while for outpatient care was negative (pro-poor) and a decreasing pattern was observed. For outpatient visits and expenses, the CI* changed from a positive sign in 2007 to a negative sign in 2011 with some fluctuations. The pro-rich inequality exacerbated for admissions while alleviated for length of stay and total inpatient expenses during the study period. The pro-rich inequality for inpatient reimbursement aggravated from 2007 to 2010 and alleviated from 2010 to 2011. For outpatient reimbursement, it altered from a positive sign in 2007 to a small negative sign in 2011. Finally, the richer needed to afford more self-payments for inpatient services and the CI* decreased from 2009 to 2011 while the inequality for outpatient self-payments changed from pro-rich in 2007 to pro-poor in 2011.

**Conclusions:**

In the NCMS, the pro-rich inequality dominated for the inpatient care while a pro-poor advantage was shown for outpatient care from 2007 to 2011 in Junan. The extent of pro-rich inequality in length of stay, inpatient expenses and inpatient reimbursement increased from 2007 to 2009, but recently between 2010 and 2011 showed a change favoring the poor.

## Introduction

Since the collapse of the old Cooperative Medical Scheme in China after the economic reforms of the early 1980s, rural residents have been excluded from the public social security system [1]. Financial barriers, among other factors, have become the most important contributor to impede the poor in trying to access medical services [1]. In order to address this problem, the Chinese government initiated New Cooperative Medical Scheme (NCMS) in 2003 to reduce the financial burden of rural residents and to achieve universal coverage.

The NCMS is organized, guided and supported by the central government but has voluntary involvement [2,3]. Unlike its predecessor (the old Cooperative Medical Scheme), it operates at county rather than village level and variations existed in design and implementation across counties [3]. The central government takes the overall responsibility to manage and supervise the scheme while the policy implementation responsibilities are decentralized to county level governments [2]. Specifically, the central and provincial governments designed the essential or basic rules about implementation of NCMS, such as the minimum level of NCMS premium and policy reimbursement rate, the priority for the reimbursement of essential drugs or Chinese traditional medicine, etc., while the county governments are responsible for specific operations, such as defining benefit packages, designating participating providers, pooling risk across the local rural population and experimenting health policy innovations like payment reform. Under this context, the benefit package was usually the same for the participants in one county or province while it may differentiate across counties or provinces, which was closely related to the varied financing levels of NCMS in different areas.

The NCMS is heavily subsidized by central, provincial and county governments and also partly financed privately from individual farmers [3]. Coverage of inpatient care is a reimbursement priority in the NCMS but also a relatively slight compensation for outpatient care, which depends on specific benefit packages in different counties. By now the scheme is also extended to cover other catastrophic diseases, such as chronic diseases, leukemia, cancer, etc. By 2011, 97.5% (around 832 million) of the rural population have been covered by the NCMS in China, meanwhile, the total NCMS revenues per capita increased from 30 RMB in 2003 to 250 RMB in 2011 (equivalent to 194 RMB in 2003 year’s price [4]), as the subsidies from governments in central, provincial and county levels rose from 20 RMB per enrollee in 2003 to 200 RMB in 2011 (equivalent to 155 RMB in 2003 year’s price [4]) (Ministry of Health, China). The rapid expansion of the NCMS inevitably raises challenging issues like any other health insurance systems as escalating healthcare costs, health care quality and the equity issue.

The socioeconomic equality in healthcare is one of the most important issues of concern in both developed and developing countries. For a specific health insurance system, it means that all enrollees should have equal access to utilize medical services, get equal reimbursement benefits and finally afford equally proportional self-payment, irrespective of their socioeconomic status, especially not dependent on the financial status [5,6]. In reality, the poor, who frequently are in need of more services, are often the least able to pay, while the wealthy utilize disproportionately more services although they have less need [7,8]. Moreover, it is also a disadvantageous factor for the poor enrollees that all individual farmers, regardless of their economic status, would pay the same contribution to be enrolled. Considering its rapid expansion and flat-rate personal contribution, it is necessary and meaningful to analyze and discuss the income related inequality situation of the utilization of medical service, reimbursement and self-payments in the current context of NCMS and more importantly, to examine the inequality changes during the evolution of NCMS.

Some studies have demonstrated the inequality status of varied aspects in the NCMS, such as benefit rate, reimbursement, medical service utilization and self-payments by enrollees [2,3,9-14]. However, contradicting results were shown across the studies and limited evidence focused on the income related inequality in outpatient care in the NCMS by using concentration index. Nevertheless, relatively fewer studies could consider the inequality changes in the NCMS by time-series data while most only concerned one or two years between 2003 and 2009. Actually, due to the new health care reform in 2009, the Chinese government stimulated more funding to be invested in the NCMS, which greatly improved its financing and reimbursement capacity. As a result, the inequality status in the current context of NCMS was unknown and rigorous empirical studies with a longer time span are strongly needed.

The aim of this study was to analyze the degree and consecutive changes of income related inequalities for both inpatient and outpatient care in the NCMS from 2007 to 2011 by using five years’ continuous data in Junan County in Shandong province, China.

The paper is organized as follows. The next section presents a comprehensive review of the most relevant literature in this field. The subsequent sections include: the description of data and methods used; the illustration of the results obtained; and the last section offers the discussions of the key findings and principle conclusions.

### Literature review

A review of the present literature concerning studies of NCMS equality could be divided into two types; one is to analyze the income related inequality among NCMS enrollees mainly by concentration index [10-14] while the others are comparing equality status between NCMS-members and non-members [2,3,9].

Regarding the first type of studies, where the equality situation among members of NCMS has been demonstrated, we mainly reviewed the studies with relatively more reliable data, larger sample and by using concentration index, the most frequently adopted method in the equality analysis. First, we concentrated on the studies using one-year data (cross-sectional studies). Considering the income related equality status in the inpatient care, the study in Mei County Shanxi Province in 2009 revealed a distinct pro-rich inequality in both inpatient benefit rate (the number of enrollees got reimbursed from the NCMS irrespective of the amount) and NCMS inpatient reimbursement [10]. Another study with data sources from three cities (Wuxi, Shengde and Chishui) in 2009 showed conflicting result that more and more inpatient reimbursement was actually concentrated on the poorer enrollees by using household data [13]. It also presented a clearly pro-rich inequality in total inpatient expenses, length of stay and self-payments [13]. The study in Yunnan Province in 2006 supported that total inpatient expenses and length of stay were concentrated more on the rich [14], in the meanwhile, it was worth paying attention in the same Yunnan study that the equality status of inpatient care utilization in the NCMS was much better than those counties without NCMS [14]. Limited evidence has been found to focus on the income related inequality in the outpatient care in the NCMS by using concentration index. The Yunnan study showed pro-rich inequality in both the number of visits and total outpatient expenses, and moreover, the degree of equality of these two indicators in the NCMS was worse than patients in the non-NCMS counties [14]. Contradictory equality result was also shown by another study that the utilization of outpatient services was concentrated more on the poorer participants, and it also revealed the pro-poor inequality in outpatient reimbursement and self-payments by using the data from three cities (Wuxi, Shengde and Chishui) [13]. Second, relatively fewer evidence could be found to reveal the equality changes in the NCMS by time-series data. The study in Jiangxi Province showed the inequality of inpatient benefit rate changed from pro-rich in 2006 to pro-poor in 2008 while the equality in the NCMS reimbursement remained relatively stable with smaller positive values of concentration index around 0.04 in all the three years by using NCMS reimbursement claim data of 40 counties in this Province [11]. In another study in Guangdong Province, the reduction of pro-rich inequality in the inpatient reimbursement among NCMS enrollees was presented by analyzing two years’ (2006–2007) NCMS reimbursement data, in the meantime, the study also showed the inequality of outpatient reimbursement changed from pro-rich in 2006 to pro-poor in 2007 [12].

In addition, three studies were found that focused on the impact of NCMS on income related inequality by comparisons between NCMS members and non-members. Particularly, by using both household survey data and routine health facility data from 15 counties in 12 provinces in 2003 and 2005, a vigorous study [3] indicated that the poor experienced larger increase in outpatient care and the rich experienced a larger increase in the inpatient care. Another two studies also supported more NCMS members from the high income group used impatient services than non-members by using data from six counties in Shandong and Ningxia provinces in 2006 [2,9].

In summary, more evidence supported the pro-rich inequality in the inpatient care while it is difficult to conclude the equality status in the outpatient care given that relatively rare studies could be found. However, we need to be cautious towards the findings revealed in these studies considering the limitations. First, the data sources used among the above studies, including both household survey and NCMS routine reimbursement data, were between 2003 and 2009 and no recent studies after 2009 related with the equality issue in the NCMS could be found according to our knowledge. Considering the development of NCMS, however, the policy priority in this study period from 2003 to 2008 (the initiation year varied among counties) was to achieve universal coverage through rapid expansion but also lower contributions. The NCMS policy priority has already changed since the new health care reform was implemented in 2009 when the Chinese government stimulated more funding to be invested in the NCMS, which greatly improved the financing and reimbursement abilities. Hence, it is necessary and more meaningful to explore the degree of income related inequality in the NCMS after 2009, more importantly, not only the equality extent at a certain time point but the consecutive changes of this inequality during a longer study period to see its evolution. Second, there were still lack of scientific evidence concerning with the inequality situation of self-payments and medical service utilization in both inpatient and outpatient care.

Given the research gap mentioned above, this paper aims to go a step further. The goal is to show the inequality status and its consecutive changes for both inpatient and outpatient care in the NCMS from 2007 to 2011 by using five years’ continuous data in Junan County in Shandong province, China. It hopes the findings could shed some light on the further evolution of NCMS and on similar health insurance systems in other developing countries.

## Data and methods

### A brief introduction of study site–Junan County

All of the data analyzed in this paper was collected in the Junan County, which is in Linyi City and located in the southeast of Shandong Province in the eastern part of China. The economic situation of the rural area in Linyi City was relatively low, ranking the 15th of all seventeen cities in Shandong Province according to the rural net income per capita 2010. The value of this indicator in the Junan County was in the last third place of the whole city [15]. The public providers of health care can be divided into three levels, village-level, town-level and county-level. The last two levels can provide both inpatient and outpatient care while the village-level can only develop outpatient services. All the public health providers are reimbursed by the NCMS. Table 1 shows the basic information of population, economy and public health providers in Junan in 2010 (the latest available data).

**Table 1:** Basic information of Junan County in 2010

**Indicators**	**Value**
**Population**	
Total	1012151
-Rural (%)	764599 (75.5)
-Urban (%)	247552 (24.5)
-Male (%)	517298 (51.1)
-Female (%)	494853 (48.9)
**Economy (RMB)**	
GDP in Junan	16175 million
Rural net income per capita in Shandong	6990
Rural net income per capita in Linyi	6761
Rural net income per capita in Junan	6665
Urban net income per capita in Junan	14908
**Health providers (the number of workers)**	
County-level	
-General hospital	1 (582)
-Traditional medicine hospital	1 (151)
-Maternal and child care service centre	1 (84)
Town-level	
-Township health centers	16 (707)
Village-level	
-Village clinics	511 (1705)

**Data source:** Population and economy data are from Linyi Statistics Bureau; health providers’ information is from baseline survey of health institutions in Junan conducted in May 2011.

**Note:** There were 18 towns in Junan from 2007 to 2009 but 14 in 2010 and 2011 since four towns were separated from Junan to construct a new economic development center under direct supervision of Linyi City. Although the official statistics of population and economy still included the four towns, the NCMS-related issues, financing, reimbursement, information system and health providers were already independent from Junan after 2009. Therefore, in order to keep our study sample consistent during the five years, only the 14 towns without administrative adjustment were included in the study.

### The principal issues of NCMS in Junan

The NCMS was launched in 2005, and by the end of 2011 it covered almost 99% of the rural residents in Junan. In Table 2 the main principles for financing and reimbursement in Junan from 2007 to 2011 are illustrated. Regarding the reimbursement procedure in Junan, the enrolled patient first need to pay all medical expenditures to the hospital at the time of the visit or admission and then get the reimbursement from the NCMS officers located in the hospital according to the expenditure claims. This arrangement is supposed to make the enrollees realizing the indeed benefit from the NCMS.

**Table 2:** The financing and reimbursement policy (in part) in Junan from 2007 to 2011

**Year**	**Financing (RMB)**		**Outpatient reimbursement**		**Inpatient reimbursement**		
	**Enrollee**	**Government**	**Rate (%)**	**Ceiling (RMB)**	**Town level (300-3000RMB) Rate (%)**	**County level Rate (%)/Deductible(RMB)**	**Ceiling (RMB)**
2007	10	40	20	NO	50	35 (200)	20000
2008	10	60	25	100	60	40 (300)	30000
2009	20	80	30	150	65	45 (300)	40000
2010	20	100	30	120	65	45 (500)	50000
2011	50	200	35	150	90	60 (500)	80000

Considering the NCMS reimbursement issue, it is necessary to understand the implications of two key terms, “reimbursement ceiling” and “reimbursement deductible”. First, “ceiling” is the highest reimbursement amount for each enrollee compensated by the NCMS in each year. For example, the ceiling of outpatient reimbursement in 2011 was 150 RMB on a one-year basis, with the meaning that the enrollee cannot receive any reimbursed further if the outpatient reimbursement in 2011 was already accumulated to 150 RMB. Second, “deductible” is always related with the utilization of inpatient services and no deductible for outpatient services. The reimbursement threshold stipulates that the enrollees could be reimbursed for the part of the expenditure exceeding the deductible and no reimbursement for the part below the deductible for every admission. For instance, assuming the total expenditure during one admission was 1700 RMB, the deductible at county hospital in 2011 was 500 RMB, as a result, for 1200 RMB, the amount exceeding the deductible (500 RMB) could be reimbursed according to the reimbursement rate (60%) and the deductible 500 RMB need to be paid by out of pocket. Generally, there is no deductible for hospitalization at town level hospitals while it is always at county hospitals.

The reimbursement principles differentiate the payment according to expenditure groups, the levels of health providers and different years. Generally, there are two basic rules. One is the level of the health institution, where a low level gives a higher reimbursement rate. The other rule concerns the outpatient service which can be reimbursed at town and village levels, but not county and county-above levels. Here, we just simplified the reimbursement principle packages and tried to give an overlook of how it was designed in Junan. For health providers at town level, the reimbursement rate for the common expenditure group (300 RMB – 3000 RMB) in all five years was listed while for county level, the reimbursement rate in the first expenditure group above the deductible was presented. For township health centers, there was no deductible from 2007 to 2009 but 150 RMB in 2010 and 2011 while the deductible for county level hospitals was much higher and shown in Table 2. Since the majority of enrollees would seek medical services within county, the reimbursement principle for the health providers outside Junan was not specified here.

Since 2007, the electronic information system has been introduced to the NCMS in Junan to track reimbursement data and help supervise the operation of designated health institutions under NCMS. It includes information of enrollees (ID, age, sex, location, etc.) and detailed expenditure records for the outpatient visits or admissions that got reimbursed by the NCMS (total expenditures, reimbursement, out of pocket expenditures (OOP), etc.). However, the information of the outpatient visits or admissions without reimbursement are not included in the system. It is probably because the enrollee did not go to the designated NCMS health providers or their expenses did not reach the reimbursement deductible.

### Data collection and study sample

In this study, we targeted all NCMS enrollees in Junan from 2007 to 2011, including data of demographic information of all enrollees and their utilization information for both inpatient and outpatient services. All the data were extracted from Junan NCMS information system. First, we constructed the dataset named “enrollees” for each year which included all NCMS enrollees with complete information. 52231 (1.4%) enrollees in total were excluded because of lack of personal information (ID, age or sex). Second, we constructed the dataset named “outpatient” in all five years which included the enrollees who utilized outpatient service and received the reimbursement. Detailed information in the “outpatient” dataset included the number of reimbursed visits, total outpatient expenses, outpatient reimbursement and out of pocket expenditures for each enrollee from 2007 to 2011. The “outpatient” dataset was then merged into the “enrollees” dataset and 3869(0.2%) outpatients in total were not matched because some enrollees without accurate information were excluded during the first step. Third, “inpatient” dataset was constructed, which included all the enrollees who utilized the inpatient services and received the reimbursement during the study period. Detailed information in this dataset contained the number of reimbursed admissions, length of stay, total expenses, reimbursement and OOP per admission. Personal information (age, sex and location) were also included for each NCMS inpatient. In this step, 8059 (3.7%) admissions in total were first excluded because of incomplete or irrational information based on three main exclusion criteria, which were (1) lack of personal information, such as age, sex, ID and address; (2) lack of admitted/discharged dates or wrong/unreasonable dates; and (3) missing expenditure data. Finally, we merged the “inpatient” dataset into the “enrollees” dataset in the second step that already contained the outpatient data and got the final study sample. After all four steps above, the final study sample included complete and accurate information. Microsoft SQL 2005, Excel 2007 and SPSS 20.0 were used to help construct the datasets. Basic information of relevant steps above is presented in the left part of Table 3 and the right part shows the sample size in all five years in the final datasets.

**Table 3:** Basic information of study sample

**Year**	**Total enrollees---all towns**			**Sample information---14 towns**		
	**Original**	**Excluded**	**Included**	**Enrollees**	**Inpatients**	**Outpatients**
2007	709900	14065	695835	559869	16635	250137
2008	790245	11551	778694	628579	31674	352644
2009	768210	9223	758987	613103	33355	415404
2010	701191	3757	697434	697434	24695	320927
2011	740485	13635	726850	726850	33674	319558

### Data analysis

#### Descriptions of analyzed indicators and methods

In this study sample, special attention was paid to analyzing the income related inequality distribution for all enrollees. The average for the following indicators was calculated for each income group. The indicators analyzed here included: (1) benefit rate, which means the share of the number of insured residents who were reimbursed by NCMS in the total number of enrollees [10], in each income group and the changes of this proportion from 2007 to 2011; (2) medical service utilization, including three indicators for inpatient service utilization(average number of admissions, average length of stay and average total inpatient expenses) in each income group, and two indicators for outpatient service utilization (average number of visits and average total outpatient expenses) in each income group; (3) NCMS reimbursement, indicating average reimbursement amount in each income group; (4) OOP, indicating average self-payments in each income group. All of the four aspects were analyzed for inpatient and outpatient, respectively. In addition, the inequality of total medical expenses, NCMS reimbursement and OOP for both inpatient and outpatient care was firstly analyzed to show the general impact in the NCMS.

Descriptive statistics and concentration index were employed to analyze the income related inequality. Descriptive methods encompassed comparisons of means, proportions or shares of the above indicators in five quintile income groups to describe the equality distribution. The concentration indices were calculated to quantify the degree and changes of income related inequality during the five years. To make comparisons consistent over time, the first year 2007 and the last year of study period 2011 were chosen for the descriptive results because they were likely to demonstrate the largest changes and the overall tendency of the five years, and the results after age-sex standardized were presented for means and shares. The values of medical expenditures, reimbursement and OOP were inflation-adjusted [4] to 2007 in order to make the comparison between 2007 and 2011. Standardized concentration indices (CI*) in all five years were presented to show the quantified results and continuous changes of the income related inequalities from 2007 to 2011.

#### Income groups

As our study was not based on the primary survey data such as household questionnaires but on the routine claim data from NCMS information system and no asset records were available for the informal sector in rural area of China, income data at individual level was not available. The annual net income per rural resident at the town level in 2010 was used as a proxy for income variable which was provided by the local health institutions during the structured health institution questionnaire in 2011 (since the survey was conducted in 2011, income in 2010 were the latest available data). Enrollees in the same town would be regarded as the same income level. Regarding the poor status of Junan County, there are small income difference within the towns for NCMS enrollees obtained by interviewing the related local officers and statisticians in Junan who were very familiar with economic status of each village in the town, but difference indeed exists in different towns, that the income of the richest town Dadian was more than doubled compared with the poorest town Laopo, this proxy can be reasonable, and this characteristics also determined to adopt the formula of concentration index for grouped data in the following analysis. Based on the income variable, we ranked all fourteen towns in Junan from the lowest annual income (4683 RMB) to the highest (10000 RMB) and calculated the number of enrollees in each town of each year, then enrollees were randomly selected from one town to the subsequent group if necessary in order to get five equally sized groups (quintile) of each year: the poorest (I), the second (II), the middle (III), the forth (IV) and the richest income group (V). The random selection was processed by SPSS 20.0.

#### Concentrative curves and the associated concentration index

**Figure 1:**
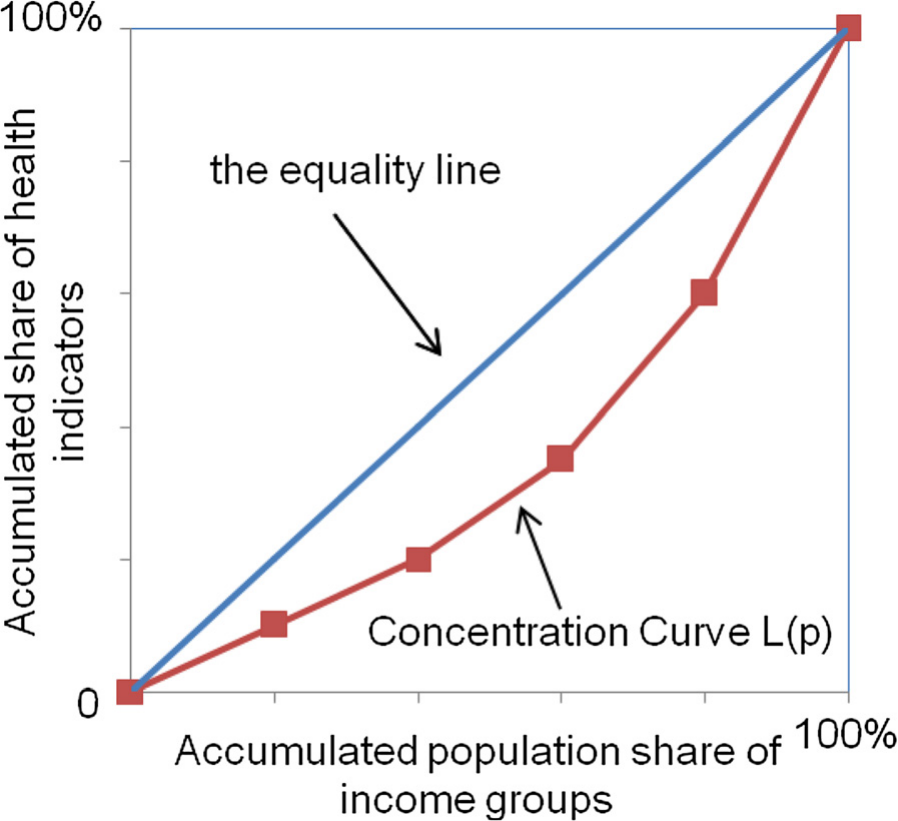
Concentration curve.

The concentration curve L(p) (Figure 1) graphs on the x-axis the cumulative percentage of the populations ranked by income beginning with the poorest, and on the y-axis the cumulative percentage of relevant indicators corresponding to each cumulative percentage of the distribution of the income variable. The concentration index aims to measure the degree of inequality in relation to income level and can be defined as twice the area between the concentration curve and the line of equality (the diagonal line) [16,17]. The convention is that the index takes a negative value when the curve lies above the line of equality, indicating disproportionate concentration of the health variable among the poor, and a positive value when it lies below the line of equality. The range of concentration index is between -1 (pro-poor) and +1 (pro-rich). Considering the data characteristics in this study, the following formula for grouped data was employed to calculate the concentration index, *CI* = (*p*_1_*L*_2_ - *p*_2_*L*_1_) + (*p*_2_*L*_3_ - *p*_3_*L*_2_) + … + (*p*_T-1_*L*_T_ - *p*_T_*L*_T-1), _where *p* is the cumulative percent of the patients ranked by income, *L(p)* is the corresponding concentration curve ordinate, and *T* is the number of income groups [16].

#### Data adjusted by age-sex standardization

In this study, the inequality caused by the income indicator is analyzed. Among other demographic factors, age and sex also play a role in generating the health inequality, which would result in the bias of equality results if raw indicators were used [18]. Therefore, the age and sex distributions need to be standardized to reduce the confounding effect of other variables as much as possible. There are two widely used techniques for the data standardization, direct and indirect methods, under the assumption that these confounding variables are correlated with health and the measure of socio-economic status [17,18]. Based on our data characteristics, direct standardization procedure is employed which is suitable for grouped data and involves applying the age-sex specific average of each income group to the age and gender structure of the sample [18]. Finally, all the analyzed indicators by income groups were standardized for ten age-sex groups (0–18, 18–34, 35–44, 45–64, and 65+) using the direct standardization method. All the results of analyzed indicators in “Results” section were after age-sex standardization.

### Ethical clearance

We got the ethical clearance from the Ethical Committee of Shandong University.

## Results

At the beginning of this section, the general status of income related inequality for total medical expenses, NCMS reimbursement and OOP for both inpatient and outpatient care was presented to reflect an overall impact in the NCMS from 2007 to 2011. Then, according to the analyzed indicators, the four aspects of income related inequality in the NCMS were illustrated for inpatient and outpatient care respectively, that is, (1) NCMS benefit rate; (2) medical service utilization, including three indicators for inpatient service utilization and two indicators for outpatient service utilization; (3) NCMS reimbursement; and (4) OOP. Both descriptive results and concentration index after age-sex standardization (CI*) are presented for each indicator from 2007 to 2011. Besides, the values and standard errors of CI* for each indicator are also shown in Table 4 (in overall) and Table 5 (for inpatient and outpatient care, respectively) in order to help obtain a thorough understanding of them.

**Table 4:** The values of CI* (standard errors) of total expenses, reimbursement and OOP in overall from 2007 to 2011

**Year**	**Overall**		
	**Total expenses**	**Reimbursement**	**OOP**
2007	0.0177 (0.0215)	0.0215 (0.0225)	0.0167 (0.0206)
2008	0.0269 (0.0191)	0.0258 (0.0192)	0.0273 (0.0674)
2009	0.0234 (0.0227)	0.0229 (0.0218)	0.0236 (0.0243)
2010	0.0207 (0.0216)	0.0240 (0.0271)	0.0273 (0.0227)
2011	0.0090 (0.0217)	0.0124 (0.0240)	0.0171 (0.0294)

Note: The values are not statistically significant by t-test (p > 0.05).

**Table 5:** The values of CI* (standard errors) for both inpatient and outpatient indicators from 2007 to 2011

**Year**	**Inpatient**						**Outpatient**				
	**Benefit rate**	**Admission**	**Days**	**Total expense**	**Reimbursement**	**OOP**	**Benefit rate**	**Visit**	**Total expenses**	**Reimbursement**	**OOP**
2007	0.0186	0.0148	0.0074	0.0293	0.0313	0.0287	-0.0211	-0.0023	0.0065	0.0114	0.0053
	(0.0270)	(0.0279)	(0.0208)	(0.0243)	(0.0213)	(0.0259)	(0.0871)	(0.0753)	(0.0372)	(0.0448)	(0.0446)
2008	0.0192	0.0197	0.0217	0.0308	0.0370	0.0282	-0.0213	-0.0110	0.0188	-0.0032	0.0256
	(0.0122)	(0.0233)	(0.0197)	(0.0257)	(0.0219)	(0.0272)	(0.0346)	(0.0769)	(0.0417)	(0.0483)	(0.0395)
2009	0.0149	0.0148	0.0206	0.0378	0.0348	0.0396	-0.0078	0.0040	0.0033	0.0012	0.0041
	(0.0316)	(0.0184)	(0.0221)	(0.0274)	(0.0248)	(0.0301)	(0.0101)	(0.0756)	(0.0531)	(0.0529)	(0.0519)
2010	0.0275	0.0299	0.0284	0.0333	0.0405	0.0308	-0.0110	-0.0083	0.0052	0.0059	0.0049
	(0.0230)	(0.0263)	(0.0282)	(0.0264)	(0.0306)	(0.0248)	(0.0196)	(0.0578)	(0.0456)	(0.0450)	(0.0422)
2011	0.0372	0.0326	0.0157	0.0185	0.0194	0.0179	-0.0105	-0.0298	-0.0115	-0.0061	-0.0140
	(0.0224)	(0.0279)	(0.0317)	(0.0322)	(0.0337)	(0.0316)	(0.0241)	(0.0612)	(0.0292)	(0.0360)	(0.0340)

Note: The values are not statistically significant by t-test (p > 0.05).

### Income related inequality of total medical expenses, NCMS reimbursement and OOP in overall

Considering inpatient and outpatient care together, as shown in Table 6, larger increases have been seen in the mean of total medical expenses, NCMS reimbursement and OOP from 2007 to 2011, especially for the growth of reimbursement. The poorest income group took the least share in 2007 for the three indicators and it accounted for the second least share in 2011. In the meantime, the richest two income groups accounted for the largest proportion in 2011.

**Table 6:** Descriptive results of total expenses, NCMS reimbursement and OOP in overall

**Income group**	**Total medical expenses**		**NCMS Reimbursement**		**OOP**	
	**Mean (RMB)**	**Share (%)**	**Mean (RMB)**	**Share (%)**	**Mean (RMB)**	**Share (%)**
**Year 2007**						
I	157.28	17.9	33.07	17.7	124.21	18.0
II	178.59	20.4	38.08	20.3	140.51	20.4
III	181.57	20.7	38.97	20.8	142.60	20.7
IV	185.94	21.2	39.90	21.3	146.04	21.2
V	173.45	19.8	37.29	19.9	136.16	19.8
**Year 2011**						
I	385.08	19.4	141.98	19.4	181.02	19.8
II	405.35	20.4	149.30	20.4	181.50	19.9
III	371.52	18.7	132.77	18.1	165.16	18.1
IV	426.37	21.5	159.62	21.8	189.54	20.7
V	399.41	20.1	149.28	20.4	198.56	21.7

**Figure 2:**
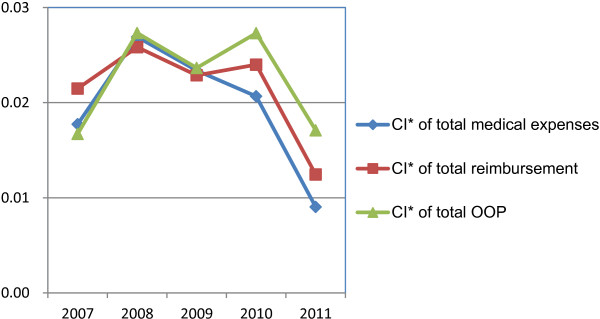
CI* of total medical expenses, NCMS reimbursement and OOP from 2007 to 2011 in total.

The CI* of total medical expenses, NCMS reimbursement, and OOP for both inpatient and outpatient care is shown in Figure 2. The inequality status and changing trends of the three indicators were almost consistent, which was all pro-rich, indicating the richer utilized more medical services, got more reimbursement and bore higher self-payments from 2007 to 2011, but the CI* decreased with some fluctuations in the study period, reflecting the extent of pro-rich was alleviated.

### Income related inequality in NCMS benefit rate

The benefit rate is used to show the percentage of enrollees who got NCMS reimbursement through inpatient or outpatient service utilization, irrespective of amount compensated. Table 7 shows the benefit rates of both inpatient and outpatient care. For inpatient care, less than 4% of enrollees benefited from inpatient reimbursement for all five income groups, especially for the two poorest income groups in 2007 while it increased in 2011 for all groups, particularly for the two richest income groups, where more than 5% of the enrollees benefited from inpatient care in NCMS. There were different changes for outpatient benefit rate. In both years, the richest group had the lowest benefit rate with no more than 40% while the middle and the second richest groups had the largest outpatient benefit rate. In addition, the benefit rate decreased for the poorest, the middle and the richest groups while a slight increase was shown for the other two income groups, which implies that the enrollees tended to utilize more inpatient service to substitute outpatient service with the development of NCMS.

**Table 7:** NCMS benefit rates for inpatient and outpatient services between 2007 and 2011 (%)

**Income group**	**2007**		**2011**	
	**Inpatient**	**Outpatient**	**Inpatient**	**Outpatient**
I	2.80	43.53	4.42	43.27
II	2.65	43.00	4.39	44.59
III	3.35	53.38	4.09	46.06
IV	3.18	43.65	5.25	46.22
V	2.89	39.74	5.06	39.57

Note: Benefit rate = the number of reimbursed enrollees/all enrollees × 100%.

**Figure 3:**
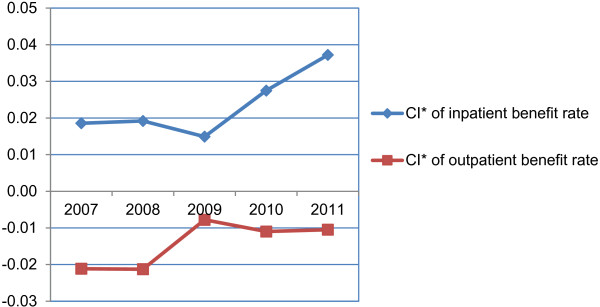
CI* of NCMS inpatient and outpatient benefit rates from 2007 to 2011.

The CI* changing trends of benefit rates are shown in Figure 3 for inpatient and outpatient care respectively. For the inpatient one, the values in all five years were positive and kept increasing except for the year of 2009, which indicates more and richer enrollees benefited from NCMS. In contrary, the negative CI* values in all five years implied a pro-poor pattern for outpatient benefits but the extent of pro-poor rate decreased gradually from 2007 to 2011.

### Income related inequality in medical service utilization

#### Outpatient visits and medical expenses

The average of reimbursed outpatient visits and medical expenses among all enrollees in 2007 and 2011 are presented in Table 8. The average outpatient visits decreased from 2007 to 2011 in all five groups, and the richest group in both years had the smallest average visits, accounting for the least proportion. There was an apparent increase in average outpatient expenses for all five groups. The middle three income groups utilized more outpatient services, representing by more outpatient visits and higher outpatient expenses in both 2007 and 2011 than the poorest and the richest groups.

**Table 8:** Descriptive results of reimbursed outpatient visits and medical expenses in 2007 and 2011

**Income group**	**No. of visits (2007)**		**No. of visits (2011)**		**Medical expenses (2007)**		**Outpatient expenses (2011)**	
	**Mean**	**Share (%)**	**Mean**	**Share (%)**	**Mean (RMB)**	**Share (%)**	**Mean (RMB)**	**Share (%)**
I	2.76	16.0	2.84	18.6	73.51	16.5	105.05	18.0
II	3.56	20.6	3.33	21.8	99.51	22.3	128.94	22.1
III	4.29	24.8	3.29	21.5	96.10	21.6	121.98	20.9
IV	4.32	25.0	3.76	24.6	98.65	22.2	131.25	22.5
V	2.33	13.5	2.03	13.3	77.39	17.4	94.63	16.2

**Figure 4:**
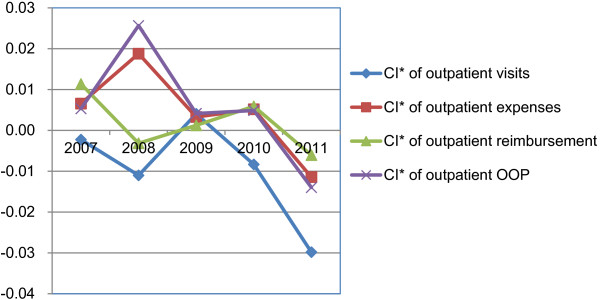
CI* of visits, medical expenses, reimbursement and OOP for outpatient care from 2007 to 2011.

The values and changing trends of CI* for the outpatient visits and expenses are shown in Figure 4. The CI* of visits were negative except for 2009 with pro-poor indication for the other four years and the pro-poor extent has increased with the NCMS expansion, from -0.002 in 2007 to -0.03 in 2011. The equality situation was reverse for the outpatient expenses, in detail, the CI* were positive from 2007 to 2010 although with fluctuating changes during this period, which indicated the rich spent more on the outpatient care than the poor.

#### Inpatient admissions, length of stay and medical expenses

Table 9 shows the descriptive results of three inpatient service utilization indicators, that is, admissions, length of stay and inpatient expenses. Compared with 2007, there were distinct increases in all three indicators in 2011, indicating all enrollees utilized more inpatient services with the development of NCMS. Furthermore, the two richest groups accounted for the largest share of all three utilization indicators in both 2007 and 2011, especially for admissions in 2011 with the total proportion of 44.4%. For the other income groups, the three utilization indictors increased slightly in the poorest income groups from 2007 to 2011 while decreased relatively more in the middle income group, which took account of the lowest share for all three indicators in 2011.

**Table 9:** Descriptive results of inpatient service utilization in 2007 and 2011

**Income group**	**No. of admissions**		**Length of stay**		**Inpatient expenses**	
	**Mean**	**Share (%)**	**Mean**	**Share (%)**	**Mean (RMB)**	**Share (%)**
**Year 2007**						
I	0.034	19.0	0.247	19.9	83.77	19.4
II	0.031	17.6	0.239	19.3	79.08	18.3
III	0.040	22.9	0.248	20.1	85.47	19.8
IV	0.038	21.7	0.252	20.4	87.29	20.2
V	0.033	18.9	0.253	20.5	96.06	22.3
**Year 2011**						
I	0.061	19.2	0.608	20.3	252.46	20.0
II	0.060	18.9	0.568	19.0	247.40	19.6
III	0.056	17.7	0.527	17.6	222.94	17.7
IV	0.073	22.9	0.680	22.7	264.60	21.0
V	0.068	21.5	0.619	20.7	276.20	21.9

**Figure 5:**
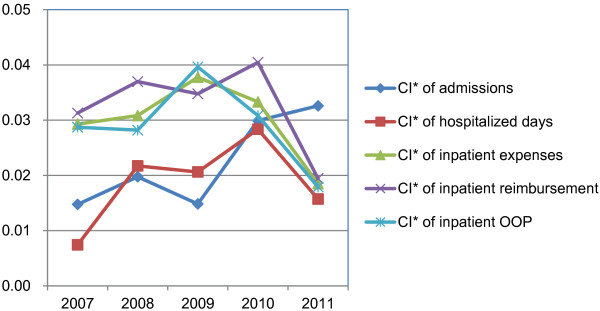
CI* of the five related indicators for inpatient care from 2007 to 2011.

The quantified results and changing trends of CI* are presented in Figure 5. The pro-rich policy message was clearly indicated by the positive values of concentration indices for all three indicators in the five years. However, they showed different changing trends from 2007 to 2011. First, the extent of pro-rich inequality aggregated for inpatient admissions although it decreased a little in 2009 but then grew up to the peak in 2011 rapidly. Second, for both medical expenses and length of stay, the CI* started to decline after 2009 and 2010 respectively, that is, the pro-rich inequality alleviated gradually in 2010 and 2011 for these two indicators.

### Income related inequality in NCMS reimbursement

Table 10 (the upper part) shows that the average NCMS inpatient reimbursement for all enrollees increased dramatically during the period from 2007 to 2011 in all five income groups. In 2007, the share of reimbursement increased when the income became higher and the richest two groups accounted for the largest share in both years. The average outpatient reimbursement doubled in 2011 compared with 2007 for all income groups. The richest and the poorest groups accounted for relatively smaller share than the other three income groups in the reimbursement of outpatient in both years. By combining inpatient and outpatient reimbursement together, it was observed that the richest group got the highest inpatient reimbursement but the lowest outpatient reimbursement in both 2007 and 2011. The reimbursement situation was disadvantageous for the poorest group that always took account of smaller share in both kinds of reimbursements in both years.

**Table 10:** Descriptive results of NCMS reimbursement and OOP in 2007 and 2011

**Income group**	**Inpatient (2007)**		**Inpatient (2011)**		**Outpatient (2007)**		**Outpatient (2011)**	
	**Mean (RMB)**	**Share (%)**	**Mean (RMB)**	**Share (%)**	**Mean (RMB)**	**Share (%)**	**Mean (RMB)**	**Share (%)**
**Reimbursement**								
I	17.97	18.9	98.84	20.1	15.10	16.4	32.98	17.7
II	17.70	18.6	97.46	19.8	20.37	22.1	41.17	22.1
III	19.24	20.2	84.28	17.1	19.73	21.4	38.98	20.9
IV	19.36	20.4	105.60	21.4	20.53	22.3	42.59	22.8
V	20.98	22.1	108.02	21.9	16.31	17.7	30.58	16.4
**OOP**								
I	65.80	19.6	153.63	20.0	58.41	16.5	72.06	18.2
II	61.38	18.3	149.94	19.5	79.14	22.4	87.77	22.2
III	66.24	19.7	138.67	18.1	76.37	21.6	82.99	20.9
IV	67.92	20.2	159.00	20.7	78.12	22.1	88.65	22.4
V	75.08	22.4	168.17	21.9	61.07	17.3	64.05	16.2

The changing trends of CI* of inpatient reimbursement and outpatient reimbursement are shown in Figure 4 and Figure 5 respectively. The positive values of CI* for inpatient reimbursement implied the NCMS reimbursement was concentrated more on the richer enrollees in all the five years. This kind of inequality kept increasing from 2007 to 2010 and decreased from 2010 to 2011 indicating the pro-rich inequality got relieved to some extent after 2010. The CI* for outpatient reimbursement presented an opposite situation, that is, the inequality changing trends were from pro-rich to pro-poor during the whole period. However, the values of outpatient reimbursement CI* were very small so the income related inequality could be regarded as pretty good from 2007 to 2011, not with distinct pro-rich or pro-poor characteristics like inpatient reimbursement.

### Income related inequality in OOP under NCMS

Table 10 (the lower part) shows average expenditures paid by enrollee themselves in each income group under NCMS and its share for inpatient and outpatient care in 2007 and 2011 respectively. Compared with 2007, self-payments in 2011 increased for all enrollees in both kinds of medical care. For inpatient services, the self-payments have more than doubled between 2007 and 2011 while outpatient self-payments rose by a relatively slight amount during the same period. The share of different income groups for inpatient self-payments increased among wealthier enrollees in 2007 and the richest two groups paid more out of pocket expenditures in both years. The proportion of inpatient self-payments accounted by the two poorest groups grew slightly by 1.6% from 2007 to 2011. Considering outpatient self-payments, the richest and the poorest income groups bore the least share in both years but the proportion of the richest declined further from 2007 to 2011 while it was reverse in the poorest by 1.7% increase which was the highest changes among the 5 income groups.

The quantified results of income related inequality in OOP is presented in Figure 4 for outpatient care and Figure 5 for inpatient care from 2007 to 2011. Overall, the inequality for inpatient self-payments was pro-rich for all five years while it changed from pro-rich to pro-poor for outpatient self-payments during this period. It means the rich paid more medical expenses by themselves but the inequality degree peaked in 2009 and then actually decreased gradually from 2009 to 2011, meanwhile, the decreasing CI* trend from 2008 to 2011 in outpatient self-payments implied the poorer enrollees afforded more and more out of pocket expenditures.

## Discussion

The NCMS have been playing a prominent role in the financial protection of rural residents by now. Over the past decade, undoubtedly, it made impressive advances towards universal health coverage with a stable participation remaining at a high level. Accompanied by huge increases in financing and reimbursement ability especially after the new health care reform in 2009, the mean values of pertinent inpatient and outpatient indicators grew rapidly as a whole, however, in the meantime, the income related equality status in certain aspects was not equalized, especially not for the inpatient care, as revealed in this study. Four key findings are worth considering further in depth.

First, the inpatient benefit rate has seen larger increases for all income groups while slight increase or even decrease was found in the outpatient benefit rate during the study period in the NCMS. The different policy reimbursement rates in the NCMS from 2007 to 2011 greatly contributed to this result. Taking the sample county Junan for example, inpatient reimbursement was priority of the government, in each year, around 70% of total NCMS contributions were allocated to the inpatient reimbursement funding while the outpatient funding only accounted for 30%. Therefore, the policy reimbursement rate for inpatient care was much higher than that in the outpatient care and moreover, from 2007 to 2011, the inpatient reimbursement rate at the town level health providers rose from 40% to 90% for the expenditure group (300–3000 RMB) while it only increased from 20% to 35% for outpatient services (irrespective of total expenditure) in the same health institutions during the same period. Consequently, more and more enrollees were stimulated to utilize inpatient services to substitute outpatient services to get higher reimbursement from NCMS, which could probably explain that the inpatient benefit rate increased dramatically from 2007 to 2011 and in the meantime no obvious changes for the outpatient benefit rate was observed. The substitution between inpatient and outpatient services was also found by Zhou [19] using the data of 2003 and 2008 national health services survey. Although his study was done in the rural area and did not target NCMS enrollees only, it is still supportive since NCMS has been the largest health insurance system in rural China covering over 95% of the rural residents by 2008. The extent and induced results of this substitution need to be carefully studied in the future, especially for the influence on medical expenses.

Second, along with the overall increase in the utilization of inpatient services, however, the distinct pro-rich inequality in the inpatient care from 2007 to 2011 was revealed in this paper, which means the poorer enrollees would not get equal benefits from the big progress achieved by NCMS. In contrast, the inequality of outpatient services was always related to pro-poor or remained relatively stable around equality line. More evidence supported the rich enrollees utilized more inpatient services than the poor [10,12,13]. Given the target of the whole rural area of China, strong pro-rich inequity of inpatient utilization still remained and income was the principal determinant of this inequality [7]. The most possible explanations for the pro-rich inequality in the inpatient care are related with NCMS reimbursement deductible and much higher medical price for the inpatient service, both of which hindered the poorer enrollees to utilize more inpatient services compared with the richer ones. In Junan, the deductible existed only in the inpatient reimbursement and it would become higher with advanced health institutions. In 2011, the deductible for town level health providers was 150 RMB while 500 RMB for county level hospitals, with the meaning that at least 150 RMB at town level and 500 RMB at county level need to be paid by enrollees themselves first before receiving NCMS reimbursement. In the meanwhile, the higher medical price of inpatient service played a vital role. In Junan, the average expenditure per admission has reached 4171 RMB by 2011 while the average expenditure per outpatient visit was only 41 RMB at the same year. Although NCMS has already made great progress in the inpatient reimbursement gradually, including the improvements of reimbursement rate and ceilings as shown in Table 2, the actual co-payment rate was still very high for the poorer enrollees, with 72.4% in 2007 and improved to 60.9% in 2011. Compared with the richer enrollees, the poorer with limited financial ability had relatively higher price elasticity and consequently, more sensitive to medical prices, which possibly lead them not to seek expensive inpatient services when the diseases are not so severe to threaten their lives in general. In contrast, the inpatient reimbursement deductible and medical price were relatively much easier for the richer to afford. NCMS released their medical demanding further and stimulated them to utilize more inpatient services to get higher reimbursement. Besides, no deductible existed for the outpatient reimbursement and the majority of the drugs prescribed by town and village level health providers were covered by NCMS reimbursement list, as a result, the poorer preferred to seek outpatient services instead.

In addition, transportation costs could also be an adverse factor for the poorer enrollees to utilize inpatient care [20,21] and stimulated them to turn to outpatient services. Enrollees could access outpatient care relatively more easily because village clinics providing outpatient care is always within walking distance. In contrary, NCMS enrollees need to go to at least the township health centers to be hospitalized, which are usually located in the center of town and usually only one in each town. The transportation costs would be much higher for utilizing the inpatient care in county or county-above health providers. Compared with outpatient care, geographical access for inpatient care would be lower.

All of them contributed to the pro-rich inequality for inpatient service utilization and more pro-poor inequality for outpatient service utilization. Since the reimbursement from NCMS is closely related with the volume and types of medical service utilization, consequently, the richer enrollees usually got higher inpatient reimbursement and also bore larger OOP under such circumstances.

Third, regarding outpatient care, it seems contradicting to find that the inequality of outpatient medical expenses was kind of pro-rich although it was pro-poor for outpatient visits and reimbursement, which was relatively distinct from 2007 to 2009. To some extent, it indirectly reflected that the rich would like to seek outpatient services from the health providers at higher level (town or county) while the poor probably inclined to visit village clinics more frequently, the lowest level of health system in China. Generally, the quality of medical care in China was closely related with the level of health providers, ie., the quality in county hospitals was always regarded as the best subsequent by the township health centers while the quality in village clinics was usually considered as the most disadvantages. Mostly, the most important function for village clinics was considered as selling drugs not giving treatment to the patients while patients could get better examinations at township health centers and county hospitals due to more skilled health personnel and advanced equipment, especially at county hospitals. The medical expenses went up higher at town and county hospitals under the same health needs compared with village clinics. As a result, although the poorer visited more frequently at village clinics, the inequality of medical expenses was pro-rich. The variation in the level of health providers the NCMS enrollees sought became the most possible explanations for the contradictory. Additionally, the location of enrollees also played a vital role as the enrollee living near the town level hospital would probably visit it for convenience instead of the village clinic.

The two aspects also can be used to explain the inconsistent changes between outpatient reimbursement and outpatient expenses in the same period. In the Junan NCMS, the ceiling for outpatient reimbursement is relatively low, as described in Table 2, and there is no reimbursement for visits at county hospitals. It would be much easier for the enrollee to reach the ceiling when seeking medical services at higher level hospitals. Thereafter, the enrollee could not be reimbursed by the NCMS for next outpatient visits that’s why the inequality of outpatient reimbursement and outpatient expenses were not inconsistent sometimes. In this study, since the outpatient visits reimbursed were merged together for each enrollee in each year, we could not quantify the influence of level of health providers and geographic access here, reminding us the further study is needed to demonstrate the inequality situation of types of health providers among NCMS enrollees.

Lastly and more recently, the development of income related inequality in the NCMS has shown a reduction in the length of stay, total inpatient expenses and inpatient reimbursement from 2010 to 2011 after its increase from 2007 to 2009, although there is still pro-rich advantage. In addition, the overall inequality trend of outpatient reimbursement changed from pro-rich in 2007 to pro-poor in 2011 with some fluctuations during the study period. Both were favorable for the poorer enrollees as they started to enjoy more equal benefits of NCMS gradually in line with the health care reform and also implied the financial access of poorer enrollees have been improved step by step. The changes were actually consistent with policy priorities of NCMS. In the initial stage, the government concentrated on rapid expansion of NCMS coverage which was necessary for a government-oriented insurance system. Under the circumstances of high copayments of inpatient utilization and limited financial ability, seeking outpatient care, instead of inpatient, became the first choice of poorer participants and a very limited demanding got released in the hospitalized services. In the meantime, the richer enrollees per se have the ability to afford the disease burden so the implementation of NCMS improved their access further. Both stimulated the increasing pro-rich inequality from 2007 to 2009. After 2009, with the implementation of health care reform, special targets and actions for NCMS were realized by improving the coverage in depth with higher reimbursement and wider benefit package, the average contribution per capita reaching 250 RMB totally in 2011 compared with 30 RMB in 2003. These policy adjustments greatly improved the policy reimbursement rate and decreased the actual co-payment rate for inpatient services at the same time. Compared with previous stage, more and more medical demands from the poorer enrollees could be released. A problem is that no later evidence or data after 2010 could be found to compare with the changing trend indicated in this study.

Regarding the pro-rich inequality in the inpatient care, which already showed reduction in the recent two years as discussed above, how to improve the inpatient service utilization among poorer enrollees and reduce current pro-rich inequality situation further are still challenging issues for the policy makers in the following stage of NCMS development. Reforming the flat-rate personal financing systems, widening the benefit package and reducing cost sharing and deductibles [21] could be probably prioritized in the NCMS policy agenda.

### Strengths and limitations

The strengths of this study could be summarized into four main aspects. In the first place, the study sample in this paper targeted the whole NCMS enrollees in one county, representing over 97% of rural residents for all five years (2007–2011) when analyzing the income related inequalities. This large sample could help make a comprehensive inequality result. Secondly, we provided the latest evidence and the continuous changing trends of income related inequality in the NCMS from 2007 to 2011, which were lacked by other studies but strongly required in practice. Thirdly, compared with other studies, more comprehensive indicators covering benefit rate, medical service utilization, NCMS reimbursement and self-payments in both inpatient and outpatient care were analyzed in this study, which could help policy makers and researchers get a better understanding of the equality status in the NCMS of China since fewer studies were found internationally published. Lastly, in this study we extracted the accurate individual utilization, reimbursement and out of pocket expenditures data from information system. Compared with other studies using household survey [9,10,13,14], the data here could be regarded as a much better accuracy because no recall bias or unconscious intension maximizing medical expenditures but minimizing reimbursement from respondents existed, which greatly contributed to a more objective conclusion. In addition, all of the data here were after age-sex standardized to avoid the confounding effects of other demographic factors besides income as much as possible, which were not mentioned in the domestic studies [11-14,22]. All of the strengths contributed to enrich our knowledge of income related inequality in NCMS.

However, the study is not without limitations. First, as we mentioned above, annual net income per rural resident at the town level was used as a proxy income variable, which would possibly underestimate the inequality in this study although the income difference within the same town for such a poor county is not so distinct. Referring to employee-based health insurance, the contribution of employees is also a useful surrogate index for actual household income because it is calculated on the income, property and private auto taxes of the employee [23,24]. For NCMS, the contribution of enrollees is flat so town-level income is finally adopted, but still income data at individual level would be preferred in the analysis of income related inequality if possible. Second, the information system included the utilizations finally got reimbursed in NCMS not all utilization information. The most possible reasons for the cases without NCMS reimbursement are that the enrollees may go to county hospitals for outpatient care or their inpatient expenses during the admission may not reach the reimbursement deductible. According to the NCMS principles, the outpatient expenses could not get reimbursed at county level and only the inpatient expenses exceeding the deductible (under ceiling) could be reimbursed. Under this condition, it could possibly make the study overestimate the average inpatient expenses and underestimate the average outpatient expenses while underestimate OOP because the excluded ones were completely paid by enrollee themselves. In addition, if the enrollee went to the non-NCMS health providers due to certain considerations (such as convenience or geographic access, etc.) when seeking medical services. They were also not reimbursed by NCMS. But the proportion of such cases should be very small due to the high coverage of NCMS health providers in the rural areas. Third, approximately 1.4% of total enrollees, 0.2% of total outpatients and 3.7% of total admissions in the five years were omitted because of lack of accurate or complete information according to the pre-determined exclusion criteria mentioned above, which was necessary for the study but also may result in certain subjective bias. In 2010, particularly, lack of admitted date or discharged date took account of 81.3% of its total missing cases. Most were hospitalizations outside the county. According to the interview of local officers, we learned that they updated their electronic information system in 2010, resulting in some merging problems which were the main reason for these missing cases. Besides, the medical service utilization and reimbursement of non-members were not recorded in the NCMS information system, so we only targeted NCMS members and did not compare the results with non-members in this study. Finally, although the paper has already standardized the age-sex distribution of NCMS enrollees to reduce the cofounding effects when analyzing income-related inequality, other important confounding factors, such as level of health providers, different deductibles and ceilings, or geographic access, also need to be considered. However, the concerning indicators were calculated for per enrollee but not per admission or visit since the goal was to analyze the inequality status from the perspective of NCMS enrollees in five years’ period. As a result, the value of health indicators was the sum of all the reimbursed visits and/or admissions occurred in each year for per enrollee. For enrollees seeking medical services more than once, the level of health providers may be different which made other confounding factors related to the choice of health providers very difficult to control. Under this context, we finally adjusted all the health indicators by age and sex.

## Conclusions

In conclusion, this study provides important suggestive evidence for the income related inequality in the NCMS by elucidating the changing trends of concentration index in both inpatient care and outpatient care from 2007 to 2011. In overall, the pro-rich income related inequality dominated in the inpatient care while pro-poor inequality was always connected with outpatient care during the study period. It is notable to find that the extent of pro-rich inequality has reduced in length of stay, inpatient expenses and inpatient reimbursement from 2010 to 2011 after the increase from 2007 to 2009. The income related inequality in the outpatient visits also changed from pro-rich to pro-poor from 2007 to 2011. However, special attention needs to be paid to the inpatient self-payments since more out of pocket expenditures started to be concentrated on the poor. Furthermore, the results revealed in this study suggest that the government should pay more attention to the equality situation of inpatient care while providing more funding to NCMS in order to make sure the poorer enrollees in need could indeed equally enjoy the benefits in medical service utilization and reimbursement from NCMS.

## Abbreviations

CI*: Standardized concentration index; CI: Concentration index; NCMS: New cooperative medial scheme; OOP: Out of pocket expenditures.

## Competing interest

All authors declare that they have no competing interests.

## Authors’ contributions

SY collected, processed and analyzed the data, wrote and finalized the manuscript. CR provided guidance and advice on the analysis framework and also detailed comments on the manuscript. XS and XL actively negotiated with local officers and participated in the data collection and paper modification. QM directed the data collection, oversaw the interpretation of data and revised the paper. All authors read and approved the final manuscript.
